# Primary lymphoma of the breast: A case report

**DOI:** 10.1016/j.radcr.2026.08.084

**Published:** 2026-09-18

**Authors:** Lembarki Sarah, El Malki Fatima Ez-Zahrae, Fatima Zahra Laamrani, Youssef Omor, Rachida Latib, Sanae Amalik

**Affiliations:** Department of radiology, Notional Institute of Oncology, CHU Ibn Sina, Faculty of Medicine and Pharmacy of Rabat, Rabat, Morocco

**Keywords:** Primary breast lymphoma, Follicular lymphoma, Case report, Immunohistochemistry, Diagnostic challenge

## Abstract

Primary breast lymphoma is a rare extranodal manifestation of non-Hodgkin lymphoma, accounting for less than 0.5% of all breast malignancies and approximately 1–2% of extranodal lymphomas. Although diffuse large B-cell lymphoma is the most frequently reported subtype, other indolent B-cell lymphomas can also occur. We present the case of a 70-year-old woman referred for evaluation of a left breast mass initially suspected to represent granulomatous mastitis on ultrasound. Histopathological examination with immunohistochemistry established the diagnosis of primary small B-cell follicular lymphoma grade 1–2 of the breast. This case highlights the diagnostic challenge posed by this uncommon entity and emphasizes the essential role of tissue biopsy and immunophenotyping.

## Introduction

Primary breast lymphoma (PBL) is a rare extranodal presentation of non-Hodgkin lymphoma, accounting for less than 0.5% of all breast malignancies and approximately 1–2% of extranodal lymphomas [1,2]. Unlike secondary breast involvement by disseminated lymphoma, PBL originates within the breast parenchyma in the absence of prior or concurrent widespread systemic disease at the time of diagnosis. The diagnostic criteria initially described by Wiseman and Liao [3] remain widely accepted and include the presence of adequate pathological material, close association between mammary tissue and lymphomatous infiltrate, and no evidence of previous extramammary lymphoma [4].

The most common histological subtype of PBL is diffuse large B-cell lymphoma (DLBCL). However, other B-cell subtypes, including follicular lymphoma (FL), marginal zone lymphoma of mucosa-associated lymphoid tissue (MALT), and Burkitt lymphoma, have also been described, although much less frequently [5]. Primary follicular lymphoma of the breast is particularly uncommon and represents a minority of PBL cases. It is generally characterized by an indolent clinical course and favorable prognosis, especially in localized low-grade forms such as grade 1–2 disease [6]. Primary follicular lymphoma of the breast is particularly uncommon, with only isolated case reports and small series published in the literature [7].

We present the case of a 70-year-old woman referred for evaluation of a left breast mass initially suspected to represent granulomatous mastitis on ultrasound. Histopathological examination with immunohistochemistry established the diagnosis of primary B-cell lymphoma of the breast.

## Case presentation

A 70-year-old woman with no medical history, referred for evaluation of a left breast mass that had been noticed by the patient approximately two months prior. Clinical examination revealed a palpable mobile mass measuring approximately 3 × 2 cm, located in the upper inner quadrant of the left breast, freely mobile relative to both superficial and deep planes with no palpable axillary lymphadenopathy.

**Fig. 1:**
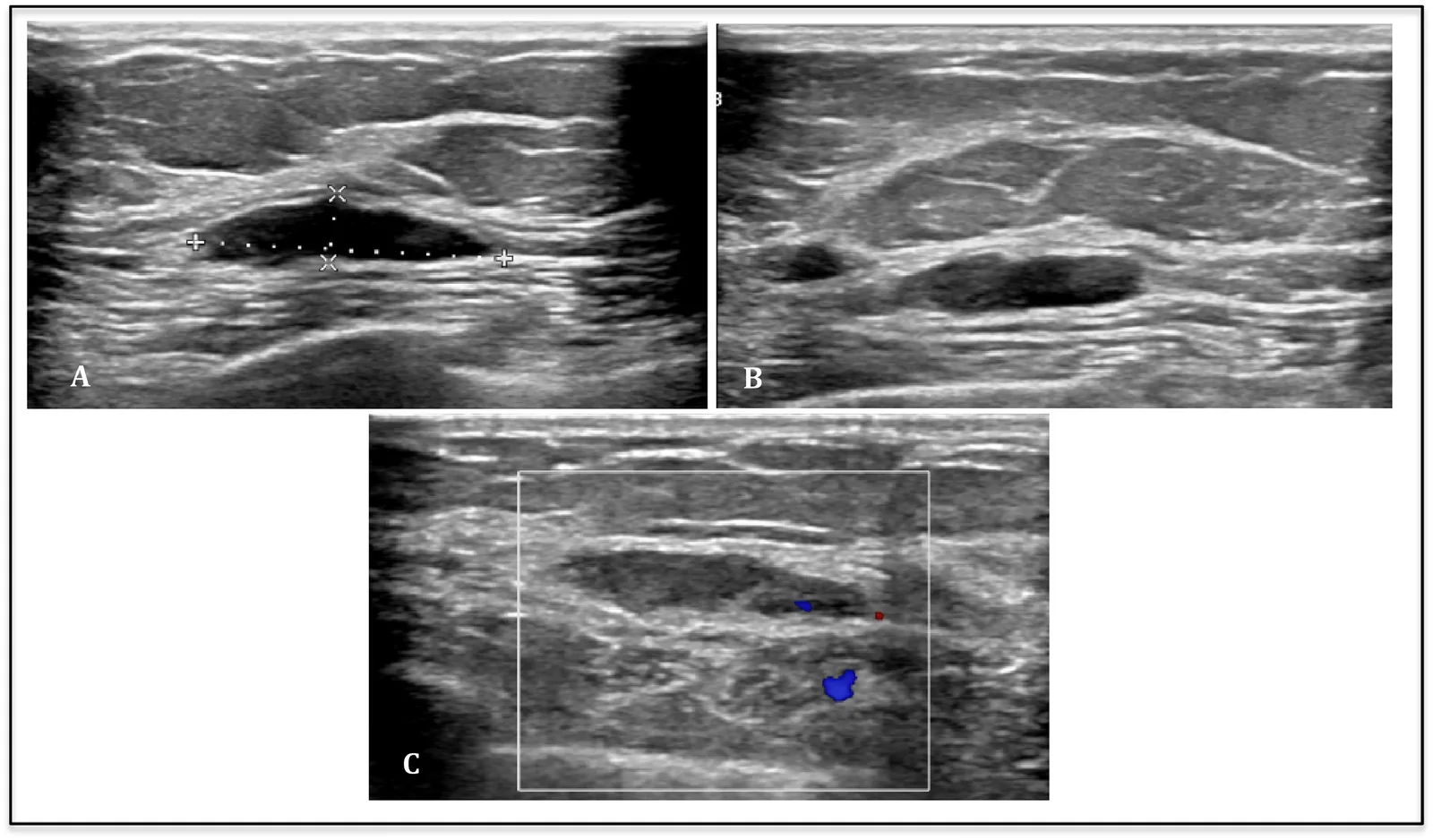
Breast ultrasound demonstrated multiple bilateral oblong masses, well circumscribed with smooth regular margins, exhibiting minimal internal vascularity on color Doppler imaging. (A) upper inner quadrant of the left breast; (B) lower inner quadrant of the right breast; (C) color Doppler.

Breast ultrasound performed demonstrated multiple markedly hypoechoic areas involving both breasts, more pronounced on the left side, initially suggestive of granulomatous mastitis. The lesion was categorized as ACR BI-RADS 4 (Fig. 1), and histological correlation was recommended.

**Fig. 2:**
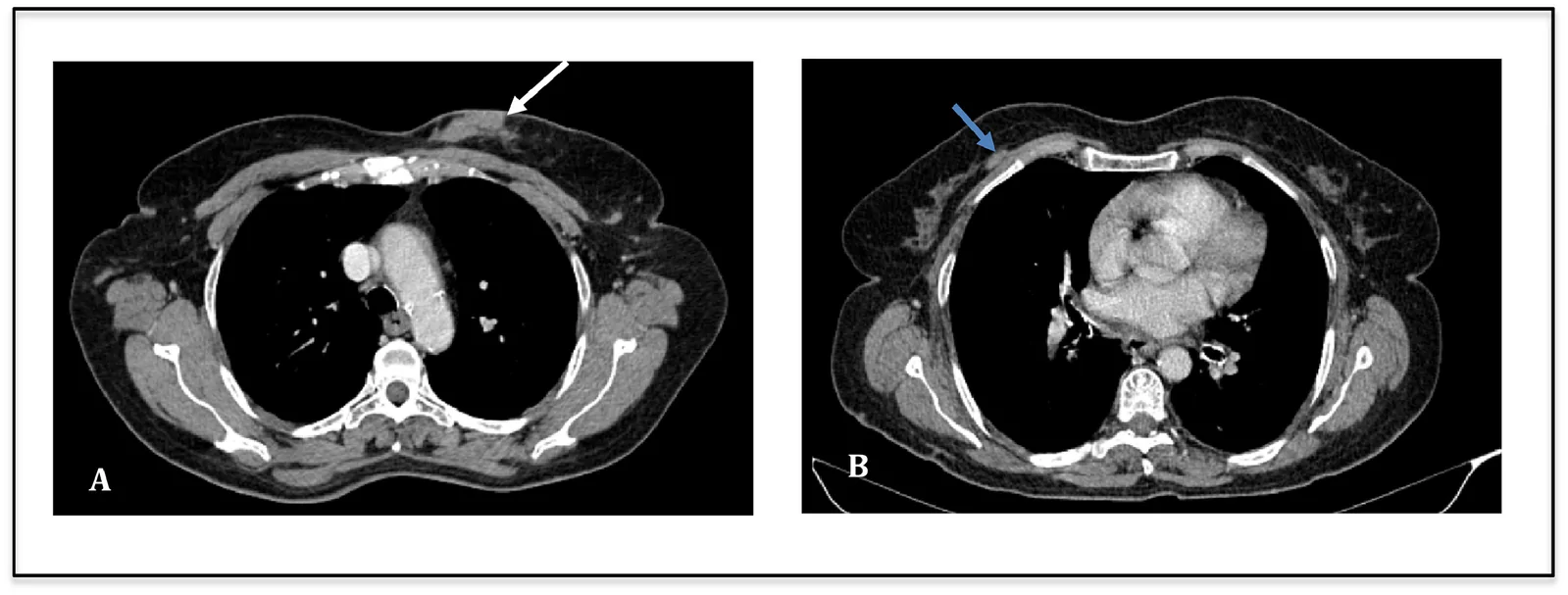
Computed tomography revealed bilateral breast masses, confluent and oblong in shape, with well-defined regular contours. (A) upper inner quadrant of the left breast (arrow white); (B) lower inner quadrant of the right breast (arrow blue).

Contrast-enhanced computed tomography of the chest, abdomen and pelvis revealed bilateral breast masses, confluent and oblong in shape, with well-defined regular contours (Fig. 2) with no lymphadenopathy and no other lesions, confirming the absence of disseminated disease.

An ultrasound-guided core needle biopsy of the left breast mass was obtained and revealed breast parenchyma infiltrated by a diffuse lymphomatous proliferation composed of small lymphoid cells, Immunohistochemical analysis showed a morphological and immunophenotypic profile consistent with a small B-cell follicular lymphoma, grade 1–2 according to the WHO 2016 classification.

Magnetic resonance imaging (MRI) of the breast was not performed, as the diagnosis had already been definitively established by core needle biopsy, and staging with contrast-enhanced computed tomography of the chest, abdomen, and pelvis had excluded associated systemic disease.

Following histological confirmation and staging workup, the patient was started on rituximab-based immunochemotherapy. Clinical and radiological follow-up is currently ongoing, with ultrasound and clinical examination planned to assess treatment response.

## Discussion

Primary breast lymphoma (PBL) is an uncommon extranodal manifestation of non-Hodgkin lymphoma. PBL primarily occurs in women, with a peak age of incidence in the sixth decade and a typical clinical presentation of a solitary, palpable breast mass [8]. The mass is typically non-tender and mobile, and less commonly it may also be associated with pain or systemic “B” symptoms [9,10]. Its presentation may also include multiple palpable masses or diffuse breast enlargement and it is rarely diagnosed on screening mammography [10,11]. which is consistent with our case

Radiologically, PBL lacks pathognomonic features. Ultrasound usually demonstrates a hypoechoic solid lesion, sometimes irregular or multilobulated, and may mimic benign inflammatory conditions such as mastitis or granulomatous mastitis, as observed in our case. This overlap in imaging findings underlines the limited specificity of breast imaging and highlights the indispensable role of tissue sampling for definitive diagnosis [12].

An unusual feature in our case was the presence of bilateral hypoechoic areas on breast ultrasound. Whether this represents bilateral lymphomatous involvement or a concomitant benign condition such as granulomatous mastitis remains uncertain, as no contralateral biopsy was performed.

Histologically, diffuse large B-cell lymphoma is the most common subtype of PBL, accounting for 40–70% of cases, whereas follicular lymphoma remains much less frequent. Several series have reported that follicular lymphoma represents only a small proportion of primary breast lymphomas, making our observation particularly uncommon [13].

The diagnosis of PBL traditionally relies on the Wiseman and Liao criteria, which include the presence of adequate pathological material, intimate association between breast tissue and lymphomatous infiltrate, absence of prior extramammary lymphoma, and no evidence of disseminated disease apart from possible ipsilateral axillary lymph node involvement [14].

From a prognostic standpoint, grade 1–2 follicular lymphoma is generally considered an indolent lymphoma with favorable outcomes, particularly in localized stages (stage I–II). Treatment usually relies on a multidisciplinary approach combining rituximab-based immunochemotherapy and/or involved-site radiotherapy, depending on stage and hematology recommendations [15].

This case highlights the importance of considering lymphoma in the differential diagnosis of atypical breast lesions, especially when imaging findings are discordant with the clinical presentation or suggest an inflammatory etiology.

## Conclusion

Primary follicular lymphoma of the breast is a rare extranodal lymphoid malignancy that may clinically and radiologically mimic primary breast carcinoma or inflammatory breast disease. Owing to its non-specific imaging features, histopathological examination with immunohistochemical analysis remains essential for establishing the diagnosis. Our case highlights the importance of considering lymphoma in the differential diagnosis of suspicious breast masses, particularly in elderly patients with atypical radiological findings. Early recognition and accurate pathological characterization are crucial for appropriate staging, therapeutic planning, and favorable patient outcomes.

## Patient consent

Written informed consent was obtained from the patient(s) for publication of this case report and accompanying images. All identifying information has been removed to ensure patient anonymity
